# Perinatal development of innate immune topology

**DOI:** 10.7554/eLife.67793

**Published:** 2021-05-25

**Authors:** Philipp Henneke, Katrin Kierdorf, Lindsay J Hall, Markus Sperandio, Mathias Hornef

**Affiliations:** 1 Institute for Immunodeficiency, Center for Chronic Immunodeficiency (CCI), University Medical Center, Faculty of Medicine, University of FreiburgFreiburgGermany; 2 Center for Pediatrics and Adolescent Medicine, University Medical Center, Faculty of Medicine, University of FreiburgFreiburgGermany; 3 Institute of Neuropathology, Faculty of Medicine, University of FreiburgFreiburgGermany; 4 Center for Basics in NeuroModulation (NeuroModulBasics), Faculty of Medicine, University of FreiburgFreiburgGermany; 5 CIBSS-Centre for Integrative Biological Signalling Studies, University of FreiburgFreiburgGermany; 6 Gut Microbes & Health, Quadram Institute Bioscience, Norwich Research ParkNorwichUnited Kingdom; 7 Norwich Medical School, University of East AngliaNorwichUnited Kingdom; 8 Intestinal Microbiome, School of Life Sciences, and ZIEL - Institute for Food & Health, Technical University of MunichFreisingGermany; 9 Institute of Cardiovascular Physiology and Pathophysiology, Walter Brendel Center of Experimental Medicine, Ludwig-Maximilians-University MunichMunichGermany; 10 Institute of Medical Microbiology, RWTH University Hospital AachenAachenGermany; Yale School of MedicineUnited States; Yale School of MedicineUnited States

**Keywords:** neonatal, immunity, innate, development, fetal, macrophages

## Abstract

At the transition from intrauterine to postnatal life, drastic alterations are mirrored by changes in cellular immunity. These changes are in part immune cell intrinsic, originate in the replacement of fetal cells, or result from global regulatory mechanisms and adaptation to changes in the tissue microenvironment. Overall, longer developmental trajectories are intersected by events related to mother-infant separation, birth cues, acquisition of microbiota and metabolic factors. Perinatal alterations particularly affect immune niches, where structures with discrete functions meet, the intestinal mucosa, epidermis and lung. Accordingly, the following questions will be addressed in this review:

How does the preprogrammed development supported by endogenous cues, steer innate immune cell differentiation, adaptation to tissue structures, and immunity to infection?

How does the transition at birth impact on tissue immune make-up including its topology?

How do postnatal cues guide innate immune cell differentiation and function at immunological niches?

## Introduction

Birth simultaneously constitutes the end of the intrauterine or fetal development, a highly complex transition, and the beginning of extrauterine life with ample environmental challenges and potential detrimental implications for long-term health. Moreover, birth converts the maternal-fetal superorganism into two, in a strictly physical sense, independent beings. The exit from the sterile womb and the establishment of metabolic independence needs to be accommodated by swift changes in virtually all organ systems of the newborn infant. Among these, the immune system inhabits a central role and undergoes broad adaptations. A precisely tuned immune system is key to a healthy life of the newborn infant since microorganisms inevitably starting to colonize during birth and thereafter need to be immediately dealt with. The postnatal phase is similarly remarkable in view of the immunological conditions, in that two individuals share their environment in a uniquely intimate fashion: immunologically active molecules are transferred via breast milk, and bacterial members of the microbiota are provided by mother to offspring. Moreover, most systemic immune effectors acquired in utero, for example, maternal antibodies, slowly wane in the infant after birth, yet maternal immune cells may circulate in the infant for a long time (called microchimerism). Thus, the immediate postnatal relationship between mother and infant maybe best termed ‘separate, but intertwined’.

Traditionally, ontogeny of the immune system has been considered to follow an upward trajectory or ‘maturation’ starting in fetal life, with the most substantial, or steep development in the first months after birth. This model has been based on the concept that, first, the fetus in utero is largely spared from microorganisms and other exogenous stressors and thus would not require classical immunity. Second, the semi-allogenic state between fetus and mother requires ‘stealth’ characteristics of the unborn child and tight immune control in the mother. In line with this, immune activation or inflammation in utero has been considered unwanted or even harmful for the unborn fetus. This has been promoted by diseases and experimental models involving pathological immune activation in utero, for example, in cases with elevated type I interferon (IFNα/β) signaling caused by congenital viral infections or in Aicardi–Goutières syndrome, which induce intrauterine growth retardation and fetal demise (Yockey and Iwasaki, 2018; Yockey et al., 2018). However, it has been a long-standing conundrum that the fetus needs to be *both*, tolerogenic in utero and prepared to instantly switch to a protective immune program with heightened local activation, when microbially colonized at birth. High complexity to the immune development before, during, and after birth manifests in distinct profiles of both activating and dampening inflammatory components at birth, for example, high counts of neutrophils, plasmacytoid dendritic cells, and tissue-resident regulatory T and myeloid-derived suppressor cells, as well as high-induced concentrations of Interleukin (IL)-10 and IL-1β (Kollmann et al., 2017; Lee et al., 2019). Global regulators – such as S100A8/A9 (calprotectin), type I IFN, or prostaglandin (PGE)-2 – have been discovered that tune immunity at the beginning of life (Ulas et al., 2017). However, these regulatory factors alone cannot explain the complexity of systemic and site-specific immune adaptation. Rather, robust systemic preparation and precise site adaptation need to go hand-in-hand to coordinate perinatal development of cellular innate immunity.

Moreover, expected perinatal disturbances, that is, infections early in life, may have long-term effects either by altering the immune cell composition or state in the different host tissues (Machiels et al., 2017) or in the bone marrow (Kaufmann et al., 2018) – with lasting effects on stem cell progeny. Alternatively, transient changes in the immune cell state may indirectly alter resident cells such as infection-driven neonatal entry of CD8+ T cells into the brain. This induces lasting epigenetic changes in microglia impacting on hippocampal function and behavior during adolescence (*Schwabenland, Henneke unpublished*).

Together, neonatal immunity is not immature, but highly regulated and specialized to best meet the unique challenges of birth and transition into the animated environment. Disentangling maternal and fetal immune development, as well as endogenous and exogenous cues around birth, requires to decipher immune cell origin at any time point of development, cellular programming, and identity imprinting by the – physiological – tissue environment, as well as adaptation to and modulation by secondary challenges, like the microbiota.

In the following, we will summarize the current knowledge and highlight the gaps in understanding the complex transitions and adaptations in the perinatal immune system and different tissues. At first, we will describe the role of immune cell-generating and -altering sites in a longitudinal track in the fetus and perinatally from yolk sac (YS), fetal liver to bone marrow. Next, we will connect in a horizontal track paradigmatic cell types and organs, that is, the skin, the intestinal tract, the lung, and the central nervous system, with respect to their perinatal makeup of local immunity. This will be complemented by describing how the blood circulation contributes to build up an effective developmentally adapted system of immune surveillance and immune cell distribution. Finally, the neonatal and the interlinked breast milk microbiota will be explored as unique cues for perinatal immune development.

In all these exemplary areas, we follow the concept that perinatal immune development comprises definable switch points to prepare for and adapt to perinatal changes of unpredictable dynamics. Perinatal cellular immune development has lasting consequences for systemic and site-specific immune regulation, including the interaction with commensal and pathogenic microorganisms. In other words, exploration of neonatal immune development sets the stage for steering immunity for a better start.

### The role of yolk sac, fetal liver, and bone marrow origin in perinatal immune cell development

The perinatal immune system comprises cells originating from distinct waves of hematopoiesis during embryonic and fetal development. In mice, these waves are characterized by a short primitive wave arising in the YS around embryonic day (E)7.5 (Palis et al., 1999), followed by a transient definitive hematopoietic wave of cells derived from erythromyeloid progenitors (EMPs) in the YS around E8.5 (McGrath et al., 2015), and the definitive hematopoietic progeny arising from emerging hematopoietic stem cells (HSCs) around E10.5 in the aorta-gonado-mesonephros region (Boisset et al., 2010).

**Figure 1.**
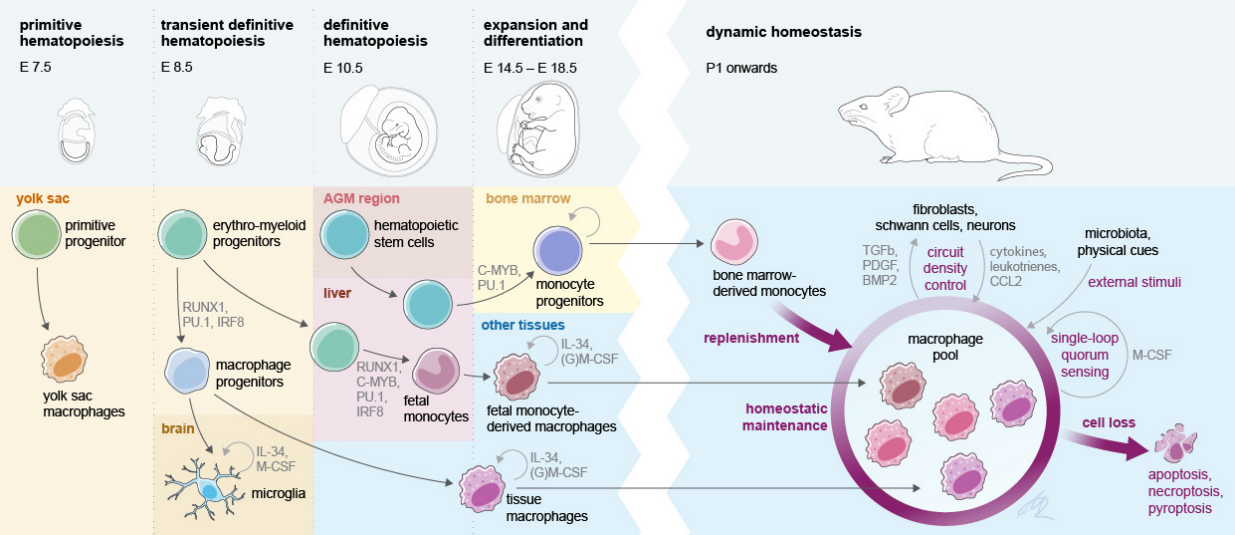
Interlinked pre- and postnatal development, adaptation, and maintenance of tissue macrophages. At birth, tissue MΦ originate from three sources that prenatally seed developing body sites in sequential waves. Postnatally, self-maintenance and renewal by bone marrow-derived monocytes are subject to microanatomical, soluble endogenous and exogenous cues. Establishment of a dynamic, tissue-specific MΦ homeostasis relies on tightly regulated late prenatal and early postnatal events.

In the last decade, a number of studies have focused on precisely defining the hematopoietic origin of various immune cells during development and on linking the emergence of immune cell populations to the different hematopoietic waves (Figure 1). As a prominent example, resident macrophages (MΦ) are seeded in host tissues during different stages of embryonic and fetal development (Goldmann et al., 2016; Guilliams et al., 2013; Hashimoto et al., 2013; Schulz et al., 2012; Yona et al., 2013). In mice, early YS EMPs and their progenitors colonize the embryo proper between E8.5 and E10.5 and seed MΦ in various target tissues (Stremmel et al., 2018). In addition, a late wave of EMPs colonizes the fetal liver and generates the first circulating fetal monocytes and granulocytes of the embryo until the first HSC-derived monocytes and granulocytes emerge (Gomez Perdiguero et al., 2015). A substantial contribution of early YS EMP-derived MΦ was described in nearly all tissues until birth. However, it seems that these cells are outcompeted in most tissues by late EMP-derived fetal monocytes, which develop in the fetal liver (Gomez Perdiguero et al., 2015; Hoeffel et al., 2015). These cells colonize the organs pre- and perinatally and then reside within most organs throughout the whole lifespan and maintain themselves via endogenous proliferation rather than continuous exchange by circulating myeloid progenitors (Hashimoto et al., 2013; Schulz et al., 2012). Thus, the fetal liver in the mouse serves as the main hematopoietic hub during development, for example, as the source of fetal monocytes but also as a side for HSC-derived progenitors until HSCs move to the bone marrow as the primary site of hematopoiesis from the perinatal period onward (Figure 1). Interestingly, also other immune cells such as mast cells or γδ T-cells in the epidermis are derived from an HSC-independent embryonic source (Gentek et al., 2018a; Gentek et al., 2018b). As noted above, EMP-derived MΦ (either from the early or late wave) persist in selected and functionally distinct tissues into adulthood, including the brain (microglia), the liver (Kupffer cells), or the epidermis (Langerhans cells). However, in several specialized tissue niches, MΦ populations are found that are indeed replaced by circulating HSC-derived progenitors from birth onwards, including the majority of dermal MΦ, lamina propria MΦ in the intestine, or stromal choroid plexus MΦ in the brain (Goldmann et al., 2016; Kolter et al., 2019; Bain et al., 2014; Van Hove et al., 2019).

Each tissue-resident MΦ population carries a transcriptional signature imprinted by hematopoietic origin (Hagemeyer et al., 2016; Lavin et al., 2014; Mass et al., 2016). Prenatally, YS EMP-derived MΦ and EMP-derived fetal monocytes compete for the same tissue niche (e.g., the alveolar space), yet fetal monocytes are transcriptionally substantially closer to adult bone marrow monocytes than to YS MΦ (van de Laar et al., 2016). As an example, EMP-derived fetal monocytes outcompete YS MΦ in the development to alveolar MΦ in late fetal stages, which is associated with the repression of the transcription factors *c-Maf* and *MafB* and a prerequisite for proper lung function and immune defense (Li et al., 2020).

Upon entry into the anatomical niche, tissue MΦ are slowly adapting to the tissue environment and a tissue-specific transcriptional program is imprinted, which is defined by many factors such as neighboring cells (Bonnardel et al., 2019; Sakai et al., 2019) metabolites, and growth factors in the niche (Okabe and Medzhitov, 2014), but also the presence or absence of microbial stimuli (Capucha et al., 2018). During the perinatal establishment of definitive hematopoiesis, immune cell progenitor cells (HSPCs) require a specific supportive environment inside the bone marrow, which regulates cell growth and retention, such as the establishment of the stem cell niche for the retention of quiescent HSCs by osteoblasts (Coşkun et al., 2014). Furthermore, endothelial cells and mesenchymal stem cells, as key sources of cytokines and stem cell factors that maintain HSPCs, appear to be essential in this context (Sugiyama et al., 2006; Hooper et al., 2009). Upon bone marrow egress, immune cells carry a developmental program, which is determined by genetic, epigenetic, and environmental cues from the stem cell niche to the target tissue. Differentiation of circulating innate immune cells, such as monocytes or granulocytes, is a hierarchical and tightly regulated process via several progenitor subsets that are instructed by defined transcriptional programs (Mildner et al., 2017; Liu et al., 2019). Furthermore, their egress from the bone marrow is tightly regulated by signals sensed, for example, via CCR2, CXCR2, CXCR4, or G-CSFR to allow a dynamic adjustment of circulating immune cell numbers during homeostasis and disease (Semerad et al., 2002; Serbina and Pamer, 2006; Eash et al., 2009). Monocytes are highly plastic cells with different subsets circulating in the blood, but are even more plastic upon entry into inflamed tissues (Guilliams et al., 2018; Giladi et al., 2020). In a recent study, even short-lived neutrophils have been shown to be highly adaptable and plastic to different tissue environment and develop distinct functional programs depending on the host tissue environment (Ballesteros et al., 2020). Proper differentiation of tissue-resident immune cells along predefined pathways is a prerequisite for tissue development and regeneration. Yet at the same time, cellular transformation processes are subject to hijacking by ‘latent’ microorganisms like mycobacteria and herpes viruses for the creation of a chronic niche (Lösslein et al., 2021).

The perinatal phase is crucial for immune cell maturation and acquisition of an immunocompetent phenotype, including tissue MΦ differentiation and adaptation to the anatomical host niche. Within their niche, MΦ undergo rapid adaptation to the changing environment and acquire often wide changes in their transcriptional landscape, as, for example, seen for the stepwise differentiation of microglia (Matcovitch-Natan et al., 2016; Thion et al., 2018). These differentiation processes are implicated both in keeping numeric MΦ homeostasis as well as a balanced response to infections (Figure 1; Kolter et al., 2019; Feuerstein et al., 2020).

It has become clear, based on multiple independent observations, that early immune cell dissemination to hematopoietic and non-hematopoietic organs is associated with important functions throughout life. However, it remains largely enigmatic, whether the effects later in life are secondary to early tissue seeding, which imprints defined programs on the tissue environment (Cossío et al., 2019). Especially around birth, immense adaptations and restructuring in the immune cell compartment are required since a variety of environmental factors get in contact with the developing organism. The origin of cells that perinatally seed tissues needs to be rigorously deciphered to resolve perinatal development of tissue immunity since early life immune seeding may have lasting consequences, that is, represents a window of vulnerability as well as opportunity.

### Perinatal adaptation of skin resident immune cells

Perinatal immune development may follow common principles, including genetic determination, in utero modulation, and postnatal adaptation, yet it may further depend on spatial and temporal signals at distinct body sites. Moreover, cellular immune development runs in parallel with the perinatal development of other cell types, for example, fibroblasts, endothelial and epithelial cells, or neurons. These cells, on the one hand, harbor restricted immunological properties, like cytokine secretion, and, on the other hand, build physical niches for immune cell interaction including residency. Thus, they reciprocally interact with transient and tissue residing immune cells and form cellular niches as – conceptionally – discrete working units for tissue immunity (Guilliams et al., 2020). In addition, MΦ renewal patterns are related to the tissue substructure, for example, highly specialized sensory nerve-associated MΦ are largely self-maintained (Kolter et al., 2019). In contrast, multicellular networks involving non-immune cells, physical cues, cytokines, stem cell factors, incoming cells, etc., tightly regulate renewal and density of the majority of tissue MΦ via only partially understood mechanisms, including quorum sensing [(Lohrmann et al., 2021) and Figure 1].

The skin is particularly interesting with respect to its highly structured microanatomy, its perinatal development, and the dynamic integration of highly diverse cues into maintaining intercellular immune cell control. In the skin, immune cell subsets of MΦ, dendritic cells, and γδ T cell lineages can be discriminated not only in view of their hematopoietic origin, but also with respect to the influence of environmental cues on cellular renewal patterns. Specifically, whereas nerve-associated MΦ in the dermis, the immune-cell-rich layer bridging subcutaneous tissue and the epidermis, and Langerhans cells (LCs) in the latter, seem to maintain their populations size in a microbiota-independent fashion (Capucha et al., 2018), the expansion and activation of other MΦ and Tγδ17 cells in the skin (Ridaura et al., 2018) is heavily influenced by the regular bacterial and fungal skin flora. Moreover, the network of professional resident immune cells in the dermis depends on the interaction with non-professional immune cells. Mechanistically, keratinocytes fortify skin barrier integrity by regulating Tγδ17 cell activation, which itself is promoted by IL-23 signals and skin microbiota (Zhang et al., 2019a). In addition, fibroblasts have the potential to produce tissue growth factors (like FGF2, FGF7, tumor growth factor β1 [TGF-β1]) in response to type I IFN, which consecutively leads to plasmacytoid dendritic cells being activated by neutrophils responding to translocating skin microbiota (Di Domizio et al., 2020).

Immune cells that are taking up residency in the target tissue have to adapt to heterogeneous tissue substructures. This process is steered by a predefined cellular program, for example, the surface expression pattern of integrins and chemokine receptors. At the same time, a large variety of cues need to be processed, such as those delivered by the extracellular matrix and non-professional immune cells in the tissue. This localization to heterogeneous tissue structures providing functional cues can result in modulation of immune responses, for example, antibacterial and anti-inflammatory functions attributed to perivascular tissue MΦ (Abtin et al., 2014; Barreiro et al., 2016). Very recently, paradigmatic evidence has linked the long-term dedication of MΦ subsets to microanatomical tissue niches and distinct transcriptomic programs. This has been exemplified for the already mentioned sensory nerve-associated MΦ of the skin (Goldmann et al., 2016; Kolter et al., 2019; Bain et al., 2014; Van Hove et al., 2019) and to MΦ localizing to the myenteric plexus in the intestinal tract (De Schepper et al., 2018). These MΦ, which function in nerve surveillance during steady state and provide cues for axon outgrowth during activation, largely keep their population size by self-renewal once they have adapted to the nerve niche. In contrast, MΦ in immediate vicinity and in the same tissue are replaced by HSC-derived progenitors, which exhibit a distinct transcriptional program (definitive hematopoiesis). Yet, upon injury and nerve outgrowth and thus niche expansion, MΦ of other origin can take up the nerve-niche specific program, independent of their origin (Kolter et al., 2019).

Epidermal LC are – due to their high antigen-presenting capacities – functionally positioned between MΦ and dendritic cells. Notably, LCs develop similar to the second immune cell lineage residing in the mouse epidermis, γδ T cells (dendritic epidermal T cells), from both early YS (YS) progenitors and fetal liver monocytes before locally self-renewing in the adult (Gentek et al., 2018a; Merad et al., 2002). The skin is colonized by EMP-derived MΦ during embryogenesis (Hoeffel et al., 2012) similar to other tissues. Starting from E14.5 onwards, EMP-derived fetal monocytes are entering the developing skin most likely via blood vessels and start to differentiate to LC progenitors (Hoeffel et al., 2012). Shortly before birth, the first LC progenitors begin to colonize the separating epidermis by transmigration from the underlying dermis into the epidermis where they peri- and postnatally differentiate to mature LCs and expand by endogenous proliferation during postnatal stages (Hoeffel et al., 2012; Chorro et al., 2009). Upon entry in the epidermis, LCs subsequently loose monocyte markers and acquire LC maturation markers including Langerin, CD11c, or major histocompatibility complex (MHC) class II. Maturation and maintenance of the adult LC population are instructed by signals of the surrounding adjacent keratinocytes, including IL-34/Csf1r signaling (Greter et al., 2012); however, the signals instructing differentiation and recruitment during perinatal development have not been identified yet. Similar to nerve associated MΦ in the dermis, the LC population is maintained by endogenous proliferation during steady state within the niche, but upon injury such as UV-B irradiation the resident LCs are eliminated and efficiently replaced by HSC-derived progenitors from the circulation (Merad et al., 2002; Seré et al., 2012). This replacement is also mirrored in part in γδ T cells in the dermis, where an IL-17-secreting subset (Tγδ17) develops before birth and is self-renewing, in contrast to IFN-γ–producing CD27+γδ T cells, which are constantly replenished by newly generated γδ-T cells from the thymus (Sandrock et al., 2018).

### Building up local immune defense in the developing lung

In the adult lung, local immune defense is mainly provided by tissue-resident MΦ. Two main sets of tissue-resident MΦ can be distinguished, interstitial MΦ (IM) and alveolar MΦ (AM) (Evren et al., 2020; Kopf et al., 2015). In the fetal and newborn mouse, the lung immune defense system is established by tissue-resident MΦ via several waves of cell recruitment (Evren et al., 2020; Kopf et al., 2015). The first wave consists of YS-derived MΦ, which can be detected in the lung from around E10. These MΦ have a heterogeneous shape with multiple pseudopods and are characterized by high expression of F4/80 and low expression of CD11b. The second wave of MΦ recruitment to the lung consists of fetal liver-derived monocytes, which appear in the lung at E12. These MΦ exhibit a round cell shape, low expression of F4/80, and high expression of CD11b (Guilliams et al., 2013; Hashimoto et al., 2013; Tan and Krasnow, 2016). Both populations are distributed diffusely in the fetal lung interstitium between E14 and 17 (Tan and Krasnow, 2016). The first wave of YS-derived resident MΦ does not substantially proliferate and remains as IM in the interstitial space. Postnatally, these cells are continuously renewed by circulating bone marrow-derived monocytes. The second wave of MΦ shows strong proliferative activity starting at E16 in the lung interstitium, leading to the establishment of the uniquely localized alveolar MΦ, which – in the mouse – start to develop around birth. In other words, fetal monocytes outcompete YS MΦ in the development to alveolar MΦ late in fetal development, which has lasting consequences for proper lung function and immune defense (Guilliams et al., 2013; Li et al., 2020; Tan and Krasnow, 2016).

The third wave of lung MΦ is recruited to the lung from bone marrow-derived circulating monocytes starting immediately after birth to fill up any niche of interstitial MΦ throughout postnatal life. Similar mechanisms concerning ontogeny of tissue-resident MΦ may also apply for the development of interstitial and alveolar MΦ during human ontogeny, as addressed in a recently published study (Evren et al., 2020).

Alveolar MΦ play a central role in the immediate postnatal protection of the lung from infections. Importantly, alveolar MΦ exhibit a unique territorial behavior with on average three alveoli being surveilled by one AM (Neupane et al., 2020). The cues for guiding alveolar MΦ function and distribution in an organ that undergoes a fourfold increase in weight in the first three weeks of life (in mice) are only partially understood. Yet, the positioning of prealveolar MΦ to the alveoli and the cell-intrinsic transition to AM appear to require the actin-bundling protein L-plastin (Todd et al., 2016). This depends on GM-CSF produced by alveolar epithelial cells (Kopf et al., 2015). GM-CSF – through binding to the GM-CSF receptor – in turn activates the transcription factor PPAR-γ, which controls the program for alveolar MΦ maturation and maintenance (Schneider et al., 2014). The unique dependence of alveolar MΦ on GM-CSF signaling among tissue MΦ in the body is illustrated by the disease alveolar proteinosis, where genetic or antibody-mediated subversion of this signaling machinery results in abnormal accumulation of surfactant-derived lipoproteins (Trapnell et al., 2019).

Alveolar MΦ keep their capacity for self-renewal throughout postnatal life (Epelman et al., 2014). Yet, they can be replaced by circulating monocytes in cases of severe damage to the alveolar MΦ population, for example, after infection or lung irradiation therapy (Machiels et al., 2017). The properties of the lung environment for alveolar MΦ function are highlighted by the ability of intratracheally transferred ectopic MΦ to adopt an alveolar MΦ phenotype (Lavin et al., 2014; van de Laar et al., 2016).

Both pulmonary development and establishment of an immune defense system in the lung are still ongoing immediately after birth. Thus, any disturbance of lung homeostasis, not only during fetal life but also in the early postnatal period, may inevitably affect lung development and local host defense. This becomes evident in premature infants, where various noxious agents or procedures (e.g., high oxygen levels, artificial ventilation, microbial products) can lead to bronchopulmonary dysplasia, a severe lung disease with long-term consequences (Blackwell et al., 2011). It is therefore instrumental to elucidate the mechanisms regulating interstitial and alveolar MΦ function in the developing lung including pathological conditions during this vulnerable postnatal time.

### Hidden immune development: the brain

The embryonic colonization by microglial progenitors in the central nervous system (CNS) and their subsequent maturation process during pre- and postnatal life is a particularly well-studied example for the development of tissue-resident immune cells. Microglia arise from the early wave of YS EMPs, and the progenitors colonize the developing brain rudiment early on during embryonic development around E9.5 (Ginhoux et al., 2010; Kierdorf et al., 2013). Once emigrated into the CNS in this early wave, the cells expand via endogenous proliferation without input of fetal monocytes or HSC-derived progenitors during later developmental stages and adulthood (Schulz et al., 2012; Hoeffel et al., 2015; Goldmann et al., 2013). With the establishment of the blood-brain barrier around E15.5, the CNS parenchyma separates from the periphery (Haddad-Tóvolli et al., 2017). In line with this separation, the microglial population is dependent on endogenous self-renewal via proliferation. Therefore, microglia show a high proliferation rate during prenatal and postnatal stages to expand and distribute in the developing CNS, but also exhibit a dramatic decline in proliferation in adult stages, once the microglial network is set up (Matcovitch-Natan et al., 2016; Thion et al., 2018). In the healthy adult CNS, the microglial entity is maintained via random proliferation with a very low turnover rate in most brain regions (Tay et al., 2017; Füger et al., 2017). Several intrinsic regulators of transcriptional programs but also exogenous cues were defined, which are involved in the stepwise maturation process of these resident immune cells in the CNS parenchyma (Matcovitch-Natan et al., 2016; Thion et al., 2018; Hammond et al., 2019; Li et al., 2019). Microglial progenitor differentiation from early EMPs in the YS relies on cell-intrinsic programs, in particular driven by transcription factors, for example, *Sfpi/Pu.1* and *Irf8* (Matcovitch-Natan et al., 2016; Thion et al., 2018; Kierdorf et al., 2013). *Sfpi/Pu.1*-deficient mice do not have any microglia in the CNS parenchyma, whereas *Irf8*-deficient mice show a reduced number of progenitors and embryonic microglia (Kierdorf et al., 2013). Adult *Irf8*-deficient mice have an altered number of microglia in the parenchyma and show severe maturation defects (Hagemeyer et al., 2016). Next to *Irf8*, the transcription factor *Sall1* was identified as a core transcriptional regulator of microglial differentiation. Loss of *Sall1* abrogates microglial development and the developing cells are highly activated and cause alterations in neurogenesis (Buttgereit et al., 2016). Moreover, microglial development is highly dependent on different cues from cells in the physiological vicinity. One outstanding example is the dependence of microglial development on colony stimulating factor 1 (CSF1) receptor signaling. Deletion or inhibition of the CSF1 receptor during development results in loss of microglia (Matcovitch-Natan et al., 2016; Thion et al., 2018; Ginhoux et al., 2010; Erblich et al., 2011). The CSF1 receptor has two ligands, IL-34 and CSF1. Both are expressed in the CNS, but – interestingly – with a spatially distinct expression pattern, which establishes postnatally (Easley-Neal et al., 2019; Kana et al., 2019). As an example, g*ray matter* microglia depend predominantly on IL-34 produced by neurons, while *white matter* microglia depend on CSF1 secreted by astrocytes during postnatal phases and adulthood (Kana et al., 2019). Of note, this distinct expression pattern of the two ligands starts to emerge around birth and establishes during postnatal phases. Besides CSF1 and IL-34, microglial expansion and homeostasis is highly dependent on TGF-β. Loss of TGF-β signaling in adult microglia results in a hyperactivated phenotype and disturbance of homeostasis (Butovsky et al., 2014; Zöller et al., 2018). Loss of TGF-β signaling during microglial development results in impairment of microglial expansion throughout the developing CNS (Utz et al., 2020). Even though microglia reside behind the closed blood-brain barrier (BBB) within the CNS parenchyma and are shielded early on during development, microglial homeostasis and development are also highly influenced by external environmental cues with one interesting example being the dependence of microglial maturation on the gut microbiota. Germ-free mice show a maturation defect of adult microglia, resulting in an altered reaction to inflammatory cues such as lipopolysaccharides (LPS) (Matcovitch-Natan et al., 2016; Thion et al., 2018; Erny et al., 2015). Therefore, microglial sensing of the microbiota seems to provide essential signals during development. Microbiota-derived signals highly affect microglia in postnatal development, whereas embryonic microglia until birth is less affected by the absence of the microbiota (Thion et al., 2018).

Microglial development is shaped by different cues derived from the CNS but also the peripheral environment. Especially during and shortly after birth manifold changes are seen in the transcriptional differentiation program of microglia. Therefore, the perinatal phase is an important time for CNS immune development and maturation. Furthermore, postnatal microglia are serving a plethora of different functions and are very dynamic in terms of mobility and proliferation, making insults in their developmental program detrimental for the CNS during this vulnerable phase.

### Perinatal adaptation of the intestinal mucosa: a timed succession of multidimensional phases to establish host-microbial homeostasis

The intestinal mucosa transits from sterility – and thus largely deprived from microbial innate immune stimuli – before birth to being densely colonized by commensal bacteria, and threatened by enteropathogens after birth. With the introduction of solid food, the intestinal mucosa is additionally subjected to a large variety of dietary antigens. The parallel exposure to food and microbiota is beneficial since microorganisms in neonatal gastrointestinal tract improve energy harvesting for growth (Ganal-Vonarburg et al., 2020). On the other hand, the challenges for the neonatal intestinal mucosa are tremendous since it has to (i) endure bacterial colonization and prevent inappropriate inflammation, (ii) direct bacterial composition of a beneficial microbiota, (iii) generate and maintain adaptive immune tolerance to dietary and microbial antigens, and (iv) establish innate and adaptive antimicrobial host reactivity to overcome challenges by enteropathogens. A timed succession of non-redundant phases facilitates postnatal immune development and the step-by-step establishment of mucosal homeostasis (Hornef and Torow, 2020). Moreover, microbiota-immune interactions starting immediately after birth have been implicated in a variety of both ‘non-communicable’ gastrointestinal diseases, like inflammatory bowel disease, as well as extraintestinal disorders ranging from psoriasis to rheumatic arthritis, metabolic syndrome, neurodegeneration, and cancer. Notably, a causal link has not been firmly established in all cases (Zheng et al., 2020).

The healthy fetal intestine is sterile, albeit low levels of microbial constituents derived from the maternal microbiota reach the neonate and contribute to early immune priming (de Goffau et al., 2019; Gomez de Agüero et al., 2016). With rupture of the amniotic membranes, the neonatal organism gets into contact with the bacteria colonizing the maternal vaginal and gastrointestinal tract (Ganal-Vonarburg et al., 2020). Whereas maternal fecal bacteria represent the dominant source in vaginally delivered neonates, C-section is associated with a more environment- or skin-like neonatal microbiota (Dominguez-Bello et al., 2010). Upon initial exposure, bacterial density quickly reaches threshold levels (van Best et al., 2020a). In contrast, bacterial diversity and compositional stability evolve slowly. During this time, dietary (breast milk versus formula feeding), genetic and environmental factors (pets, siblings, geography), as well as medical interventions (antibiotics, probiotics) influence microbial composition (van Best et al., 2020b; Bokulich et al., 2016; van Best et al., 2015). In general, the microbiota built-up follows programmed succession of highly abundant bacterial species (van Best et al., 2020a; Planer et al., 2016; Feng et al., 2020). In humans, it requires 2–3 years after birth for bacterial diversity and composition to reach adult levels and stability (Bäckhed et al., 2015; Yatsunenko et al., 2012). Whereas microbial antigens are already present in the intestinal lumen at high concentrations early after birth, direct exposure to dietary non-self antigens starts later, in particular with the introduction of solid food. Neonatal sepsis and meningitis caused by bacterial pathogens, such as *Streptococcus agalactiae,* occur early and represent an important cause of neonatal mortality worldwide (Kolter and Henneke, 2017). In contrast, only specific viruses, for example, cytomegalovirus contaminating breast milk and respiratory syncytial virus, infect infants already shortly after birth, whereas the bulk of viral infections occur later with increasing human contact and the cessation of maternal protection by transferred IgG and SIgA (Ershad et al., 2019; Baasch et al., 2020).

An essential issue in this context relates to the specific mechanisms that allow the neonatal intestinal mucosa to accommodate microorganisms and their potentially highly activating effector molecules. Both the intestinal epithelium and resident immune cells express innate immune receptors such as Toll-like receptors (TLRs), Nod-like receptors (NLRs), RIG-I-like helicases, and thus sense microbial stimuli and secrete chemotactic and proinflammatory mediators. Age-dependent differences in the expression levels of innate immune receptors may regulate immune cell activation at this critical time point (Pott et al., 2012; Gribar et al., 2009). In addition, adaptive mechanisms adjust cellular reactivity in order to maintain tissue integrity. For example, shortly after birth the epithelium undergoes reprogramming and thus acquires innate immune tolerance via miR-146a-mediated translational repression of the TLR signaling molecule IL-1 receptor-associated kinase 1 (Lotz et al., 2006; Chassin et al., 2010). At the same time, immune cells undergo reprogramming via the endogenous alarmin S100A8/A9 interacting with TLR4 (Ulas et al., 2017). Other regulatory molecules, for example, A20, single immunoglobulin IL-1 receptor-related molecule (SIGIRR), IL-1 receptor-associated kinase-M, and Toll-interacting protein (TOLLIP), contribute to controlling the activation state of the intestinal cells (Vereecke et al., 2010; Nanthakumar et al., 2011). Furthermore, molecules such as IL-10 or arginase 2, secreted, for example, by neonatal B cells and CD71-positive erythroid cells, as well as the immunosuppressive properties of amniotic fluid and breast milk constituents, may provide synergistic cues in this context (Zhang et al., 2007; Elahi et al., 2013; Good et al., 2012). Notably, the ability to control inappropriate innate immune stimulation depends on the organism’s gestational age. Necrotizing enterocolitis (NEC), which is mainly observed in preterm infants and is associated with severe colonic inflammation, is thought to result from a failure to control microbiota-induced immune stimulation (Neu, 2020; Gopalakrishna et al., 2019). Currently, specific causal microorganisms have not been identified. However, frequent alterations in the microbiota of the preterm infants, and here in particular an elevated abundance of members of the phylum proteobacteria, are likely to be significant. Accordingly, oral administration of probiotic Bifidobacterium and Lactobacillus spp. modifies the microbial composition in these children and lowers the incidence of NEC and sepsis (van Best et al., 2020b; Neu, 2020; Panigrahi et al., 2017).

The fact that maternal fecal bacteria represent an important source for the infant's microbiota ensures the transmission of a usually ‘successful’ microbiota to the next generation. Delivery mode impacts newborn gut colonization efficiency (preprint BioRxiv. doi: https://doi.org/10.1101/2020.01.29.91999). In addition, the newborn infants actively shape a beneficial microbiota composition. For example, and as outlined in detail below, human milk oligosaccharides (HMOs) are highly abundant complex carbohydrates that are indigestible for humans. They foster the growth of Bifidobacterium, the hallmark bacterium of the healthy infant microbiota (Zivkovic et al., 2011). In mice, hepatic secretion of bile acids into the proximal small intestine promotes the expansion of lactobacilli and accelerates microbiota maturation (van Best et al., 2020a). Finally, specific expression of the flagellin receptor TLR5 by the neonatal gut epithelium favors colonization by non-flagellated bacteria in comparative colonization and microbiota transfer experiments (Fulde et al., 2018). These and other mechanisms might foster regular development of the neonatal microbiota-intestine interface to counteract predictable disturbances, such as infections (Pop et al., 2014).

Despite early homing of lymphocytes and antigen-presenting cells to the mucosal lymphoid tissue, the murine adaptive immune system appears to underlie active suppression during the first two weeks of life (Torow et al., 2015). This may allow establishment of adaptive immunity, while – at the same time – avoid autoimmunity. The cessation of breastfeeding causes significant alterations of the microbiota composition and induces goblet cell-associated antigen passage (Kulkarni et al., 2020). It causes the so-called ‘weaning reaction’, which leads to a transient innate immune stimulation, including accelerated replacement of fetal MΦ in the lamina propria by monocyte-derived MΦ, and the initiation of adaptive mucosal immunity (Al Nabhani et al., 2019). This reaction propagates the maturation of regulatory T cells and thus the maintenance of immune tolerance (Kulkarni et al., 2020; Al Nabhani et al., 2019; Yang et al., 2015; Vatanen et al., 2016). Only then effector T lymphocytes are generated that provide antimicrobial protection. However, these early T lymphocytes derive from separate progenitors and exhibit significant differences to their adult counterparts (Mold et al., 2010). Their unique properties such as rapid effector responses, but reduced ability to form memory, match the requirements of neonatal life, such as limited metabolic resources (Rudd, 2020).

Infants provide efficient antimicrobial responses to control pathogen challenge despite the described mucosal immune restrictions, First, exogenous antimicrobial factors such as lactoferrin, antimicrobial peptides, and SIgA in breast milk support pathogen resistance. Also, the neonatal epithelium appears to discriminate between microbial colonization and invasive infection via the formation of chemotactic and antimicrobial mediators (Zhang et al., 2014; Dupont et al., 2016). Finally, newborn infants in the event of viral infection are able to mount a fast and effective cellular immune response although the emerging T lymphocytes are more prone to rapid effector function than lasting memory function (Pott et al., 2012; Sarzotti et al., 1996). Much needs to be learnt to improve understanding of the functional and structural particularities of the neonatal intestinal mucosa, which may form the basis for improved preventive and therapeutic strategies at the beginning of life.

### Breast milk and gut microbiota are intertwined regarding their impact on perinatal immune development

Breast milk is a complex cocktail of biologically active components and nutritional substrates with substantial compositional differences between individual mothers and changing over time of lactation (i.e., between colostrum and mature milk). Key components such as immunoglobulins, chemokines/cytokines, bioactive micro- and macronutrients, oligosaccharides, microRNAs, hormones, immune cells, and microorganisms have been associated with direct and indirect perinatal immune programming.

HMOs comprise ~200 different bioactive glycans. They contain direct immune-mediated effects, such as antiadhesive, antimicrobial, and immune programming activity (Walsh et al., 2020). HMOs promote immune cell homeostasis and reinforce intestinal epithelial barrier integrity (Xiao et al., 2019; Šuligoj et al., 2020; Zhang et al., 2019b). Consistently, the concentration of the HMO disialyllacto-N-tetraose is inversely correlated with the infant’s risk for NEC (Masi et al., 2020). In addition, there is limited data suggesting that HMOs circulate in the maternal blood throughout gestation and may cross the placenta (Hirschmugl et al., 2019; Wise et al., 2018). However, the functional relevance of these findings for mother and fetus remains to be explored. In addition, HMOs act as prebiotics promoting the growth of beneficial bacteria that in turn impact on the mucosal and systemic immune system. HMOs are poorly metabolized by the host, but are favored growth substrates for certain beneficial Bifidobacterium species (Fulde et al., 2018). Immune-modulatory effects may occur through recognition of cell surface-associated microbial components including bifidobacterial exopolysaccharides and tad pili, and the production of immune-modulatory metabolites, for example, short chain fatty acids (SCFAs) (Alessandri et al., 2019). Strikingly, an increased Bifidobacterium abundance in the human and mouse gut was described already during pregnancy. Thus these effects may start prior to birth *via* placental crossing of microbial metabolites and in utero programming (Nuriel-Ohayon et al., 2019). It remains unclear, however, how specific strains of Bifidobacterium and other early life microbiota constituents and their metabolites trigger immunomodulatory responses that maintain immunological homeostasis during the perinatal period.

Breast milk and, in particular, colostrum provide other micro- and macronutrients with high immune-modulatory activity, such as amino acids, lipids, vitamins, and trace elements (Dror and Allen, 2018). Specific amino acids like glutamate and glutamine (the most abundant free amino acids) are essential for immune and tissue development (Dror and Allen, 2018). The second largest nutrient fraction comprises lipids, including the long-chain poly-unsaturated fatty acids arachidonic acid and docosahexaenoic acid. These have been shown to influence lymphocyte proliferation and cytokine production. In addition, iron (bound to lactoferrin) and zinc are central in perinatal immune development and may act indirectly via growth modulation of specific gut bacteria. Importantly, obese or malnourished mothers may potentially lack an appropriate balance of immune-modulatory components impacting perinatal immune programming in their infants (Samuel et al., 2020). Also, the direct transfer of immune cells, such as T cells, NK cells, and MΦ, via breast milk may influence perinatal immune development (Laouar, 2020). Whereas it remains unclear whether intact immune cells can translocate through the infant’s mucosal barrier and populate neonatal tissues (Torow et al., 2015), recent work on CD8+ memory T cells indicates that even cellular lysate may still represent a mechanism for passive transfer of cellular immunity (Myles and Datta, 2021). Moreover, stem-cell-like cells in breast milk have high multilineage potential and can traffic to different body sites including the brain. Yet, whereas their differentiation potential into neuronal and glial cell types has been demonstrated, it remains unclear whether they can also differentiate into immune cells (Ninkina et al., 2019). Breast milk also contains exosomes enriched in immune-modulating proteins, peptides, and miRNAs (e.g., miR-30d-5p, let-7b-5p, and let-7a-5p), which seem to withstand digestion and may promote intestinal epithelial cell growth and mucosal barrier development (Hock et al., 2017). Other directly immunomodulatory components include immunoglobulins (Ig), with secretory (S)IgA being dominant (>90%), followed by SIgM. IgG in humans is mainly found in mature milk. SIgA carries major antimicrobial properties, but recent data suggest an additional role of SIgA in *enhancing* bacterial colonization and homeostatic immunity (Cacho and Lawrence, 2017; Pabst and Slack, 2020). T cell help is required to generate appropriate affinity maturation to bacterial antigens including antigens from segmented filamentous bacteria (Kawamoto et al., 2012). Growth factors and cytokines such as EGF, TGF-β, or the S100 calcium binding proteins are passed from mother to infant via breast milk and modulate mucosal epithelial and immune responses (Kociszewska-Najman et al., 2020). This transfer is influenced by birth mode and/or gestational age. For example, preterm infants exhibit lower levels of fecal S100 proteins (i.e., fecal calprotectin), leading to changes in the gut microbiota, including alterations in the abundance of members of the Bifidobacteriaceae family (Willers et al., 2020). Breast milk also contains microorganisms such as Staphylococcus, Streptococcus, as well as low numbers of Bifidobacterium and Lactobacillus species (Demmelmair et al., 2020; Cortes-Macias et al., 2020). An associated influence of immune development may occur through bacterial metabolites, such as SCFAs. These modulate immune cell activity, for example, via the inhibition of histone deacetylases and the activation of G protein-coupled receptors. Butyrate at concentrations found in breast milk modulated immune responses in an in vivo food allergy model (Paparo et al., 2020).

The enteric microbiota impacts on the development and function of resident immune cells at mucosal sites but also well beyond mucocutaneous surfaces, for example, in the CNS (Oschwald et al., 2020; Elinav et al., 2011). Tissue-resident MΦ seeded in embryonic life are postnatally replaced by bone marrow, monocyte-derived MΦ in a highly tissue-specific fashion (Lohrmann et al., 2021). This process is influenced by the acquisition of the microbiota (Bain et al., 2014) or can result from low-grade infections, for example, by herpes viruses in case of alveolar MΦ (Machiels et al., 2017). Notably, the comparison of SPF mice with germ-free or antibiotic-treated mice may underestimate the contribution of the endogenous microbiota since SPF mice are relatively close to GF mice and distant to mice with a more diverse, ‘natural’ flora (Rosshart et al., 2019). In contrast, the role of the microenvironment including the microbiota on perinatal development of non-professional immune cells that contribute to immune niches, like fibroblasts and epithelial cells in the skin, has not been sufficiently taken into consideration. In addition, the impact of the maternal microbiota on the placenta as an important fetal immune organ has remained largely elusive.

### Blood circulation and immediate immune responses

During fetal ontogeny, various immune cells populate peripheral organs and install a broad defense network to gradually prepare for the challenges after birth. The distribution of immune cells and their precursors throughout the organism relies on effective transportation by the blood circulation. In the mouse fetus, the onset of blood circulation is observed at around E8.5 and is fully established at around E10.5 (Cacho and Lawrence, 2017). During this time, the first monocytes and neutrophils derived from EMPs of the YS are released into the blood circulation (Stremmel et al., 2018; McGrath et al., 2003). At later time points, they are replaced by neutrophils/monocytes derived from HSCs originating from sites of intraembryonic definitive hematopoiesis, which takes place first in the fetal liver and then gradually moves to the bone marrow. While early fetal circulating monocytes have been identified to seed peripheral organs and differentiate into tissue-resident MΦ, as described above, the fate and function of early circulating neutrophils are less clear. Yet, several lines of evidence support the hypothesis that circulating neutrophils already provide the first line of immediate immune response, just as in postnatal life.

During fetal life, neutrophils exhibit an ontogenetically regulated gain of recruitment efficiency into inflamed mouse YS vessels, as revealed by intravital imaging. The scrupulous analysis of neutrophils from preterm and term infants after birth showed that fetal as well as postnatal neutrophil development follows a cell-intrinsic functional program, which intriguingly does not seem to be significantly altered by cues from early postnatal life (Sperandio et al., 2013; Nussbaum et al., 2013). This indicates that the ontogenetic neutrophil-specific program is tuned to perfectly adapt to the specific needs and challenges of the growing organism within the uterus leaving room for development and at the same time provides enough protection against exogenous threats. However, adaption of neutrophils to premature postnatal life is rather limited. Paucity and altered activation of neutrophils have been traditionally linked to infection susceptibility and high-related mortality in newborns, especially preterm infants (Gessler et al., 1995; Henneke et al., 2003).

As compared to granulocytes, induced activation of circulating mononuclear phagocytes , in particular monocytes and dendritic cells, from newborn infants has been subject to rather detailed analysis. The results point to complex and dynamic alterations as compared to similar cells from older children and adults. Overall, mononuclear phagocytes from cord blood, that is, late fetal cells at the transition to independent postnatal life, show robust responses to whole bacteria and purified bacterial effectors with respect to some cytokines (e.g., TNFα, IL-6, and IL-10), yet respond weakly with type I interferons to cytomegalovirus infection (Kollmann et al., 2017). Unique properties of cord blood mononuclear cells as compared to those from young infants in terms of cytokine formation have been confirmed by high-dimensional analysis (Olin et al., 2018). Yet, the functional implications of high activation at baseline and after stimulation around birth remain largely enigmatic.

## Summary and outlook

Distinct immune cell niches exist in complex tissues and undergo major adaptations and changes during the perinatal period. They can be discriminated by distinct cellular programs. Both systemic regulators and microenvironment-specific signals steer homing and homeostatic maintenance of resident immune cells by transcriptionally redirecting progenitors from different sources.

The impact of exogenous signals,such as the establishing microbiota and microbial metabolites as well as tissue-intrinsic factors, such as the perinatal change in oxygen tension on local cell composition, cell differentiation, and immune maturation and the interplay between local and systemic regulatory mechanisms are largely elusive. Understanding of these issues is pivotal, since perinatal changes set the stage for tissue adaptation, immune homeostasis, and long-term health and may therefore facilitate the development of interventional strategies to prevent disease disposition during this critical period.
